# Renal Tissue Oxygenation in Essential Hypertension and Chronic Kidney Disease

**DOI:** 10.1155/2013/696598

**Published:** 2013-02-20

**Authors:** Menno Pruijm, Lucie Hofmann, Bruno Vogt, Marie-Eve Muller, Maciej Piskunowicz, Matthias Stuber, Michel Burnier

**Affiliations:** ^1^Department of Nephrology and Hypertension, CHUV, Rue du Bugnon 17, 1011 Lausanne, Switzerland; ^2^Department of Radiology, Medical University of Gdansk, Gdansk, Poland; ^3^Department of Radiology, CHUV, Lausanne, Switzerland

## Abstract

Animal studies suggest that renal tissue hypoxia plays an important role in the development of renal damage in hypertension and renal diseases, yet human data were scarce due to the lack of noninvasive methods. Over the last decade, blood oxygenation level-dependent magnetic resonance imaging (BOLD-MRI), detecting deoxyhemoglobin in hypoxic renal tissue, has become a powerful tool to assess kidney oxygenation noninvasively in humans. This paper provides an overview of BOLD-MRI studies performed in patients suffering from essential hypertension or chronic kidney disease (CKD). In line with animal studies, acute changes in cortical and medullary oxygenation have been observed after the administration of medication (furosemide, blockers of the renin-angiotensin system) or alterations in sodium intake in these patient groups, underlining the important role of renal sodium handling in kidney oxygenation. In contrast, no BOLD-MRI studies have convincingly demonstrated that renal oxygenation is chronically reduced in essential hypertension or in CKD or chronically altered after long-term medication intake. More studies are required to clarify this discrepancy and to further unravel the role of renal oxygenation in the development and progression of essential hypertension and CKD in humans.

## 1. Introduction

Hypertension is a major public health burden and affects about 1 billion people worldwide. Hypertension increases the risk of coronary heart disease and cerebrovascular accidents [1, 2]. Hypertension is also tightly entangled with chronic kidney disease (CKD) [3, 4], defined as “evidence of structural or functional kidney abnormalities that persists for at least three months, or a glomerular filtration rate (GFR) < 60 mL/min/1.73 m², with or without evidence of kidney damage, and irrespective of its cause” [5]. A majority of CKD patients are hypertensive, and hypertension is one of the main causes of CKD, accounting for approximately 30% of cases of end-stage renal disease (ESRD) [6, 7]. Hypertension and CKD cluster in obese and/or diabetic subjects, and both disease states share common underlying pathways, such as chronic low-grade inflammation, increased sodium retention, and an activated renin-angiotensin system [8, 9]. The kidneys are seen by some as the main culprit in the development of hypertension. Transplantation studies have indeed demonstrated that hypertension follows the kidneys [10, 11], and in extreme cases removing both kidneys in transplanted patients can cure resistant hypertension [12]. 

Animal studies have suggested that renal tissue hypoxia might be another common mechanism of renal damage in hypertension and renal diseases [13]. Indeed, cortical and medullary tissue hypoxia has been documented in both disease states [14, 15]. However, translation of animal studies to humans should be made cautiously and is hampered by the absence of non-invasive techniques allowing the measurement of renal tissue oxygenation in humans. This paper focuses on the determinants of renal tissue oxygenation in humans and on the actual evidence pointing towards a role for renal tissue hypoxia in the development and progression of hypertension and CKD in humans. 

## 2. Determinants of Renal Tissue Oxygenation

Fifty years ago, Aukland and Krog were the first to report the unique oxygenation pattern of kidneys using microelectrodes sensitive to oxygen in dogs and rats. They measured levels of tissue partial pressure of oxygen (pO_2_) as low as 10–20 mmHg in the medulla and 50 mmHg in the renal cortex [16], despite the fact that renal blood flow, and thus oxygen delivery, is the highest in the body in relation to organ weight. Since then, much has been learned on the mechanisms involved in the regulation of renal medullary tissue oxygenation. As in any organ, the local tissue pO_2_ is determined by the oxygen delivery (a function of renal perfusion and blood oxygen content) and by the O_2_ consumption, which is driven essentially by the glomerular filtration rate and the active tubular transport. Renal kidney perfusion is characterized by preglomerular oxygen diffusion shunting, partly explaining the low tissue pO_2_ levels [17]. In the kidneys, over 90% of all oxygen consumption is used for tubular sodium transport, which differs between cortex and medulla. The well-perfused proximal tubules are mainly located in the renal cortex. Proximal tubular sodium reabsorption is partly based on energy-consuming active transport via basolateral Na^+^, K^+^-ATPase and partly on passive transport via paracellular pathways [18]. Hence, despite the fact that the proximal tubules reabsorb 67% of all filtered sodium, they consume only 27% of total O_2_, resulting in a cortical pO_2_ around 50 mmHg [19–21]. In contrast, Henle's loops in the medulla receive 10% of RBF and reabsorb 30% of sodium at a relatively high-energy cost. They use 67% of all O_2_, and local pO_2_ is as low as 10–15 mmHg under normal circumstances, which renders them highly susceptible to ischemic injury [19, 20].

Theoretically, worsening of renal tissue oxygenation is the consequence of reduced oxygen delivery or of increased oxygen consumption. Examples of the first category are severe renal artery stenosis, renal hypoperfusion due to septic or cardiogenic shock, or severe anemia. Examples of the second category are energy-consuming glomerular hyperfiltration as seen in obesity and the early stages of diabetes, proinflammatory pathways, and/or enhanced renal sodium reabsorption, as present in essential hypertension. 

Whatever the cause, the consequence of renal tissue hypoxia is identical, that is, activation of hypoxia-induced factors (HIF-1) and HIF-1 regulated genes. The activation of these genes will not only lead to an increased erythropoiesis and to neoangiogenesis through activation of vascular endothelial growth factor and inducible nitric oxide synthase, but also to the stimulation of growth factors that cause endothelial dysfunction, activation of inflammatory cells, and oxidative stress, which will lead to tissue injury and interstitial fibrosis [22, 23]. Whereas there is accumulated evidence for a role of hypoxia in the development of acute and chronic renal damage in animals, there is definitively less information on the pathophysiological role of renal hypoxia in humans. This is due essentially to methodological reasons. Indeed, many techniques in animals allow measurement of regional renal oxygen content, such as the insertion of oxygen microelectrodes and the protoporphyrin phosphorescence method [24]. Besides, hypoxia can be measured on dissected kidney tissue using pimonidazole, a molecular “hypoxia probe” which, following injection, binds to tissues with oxygen tension below 10 mm Hg [15] or by tissue dosing of HIF-1 or hypoxia-responsive genes. Unfortunately, these techniques are too invasive or not adapted to be used in humans.

## 3. Measurement of Renal Tissue Oxygenation in Humans with BOLD-MRI

**Figure 1:**
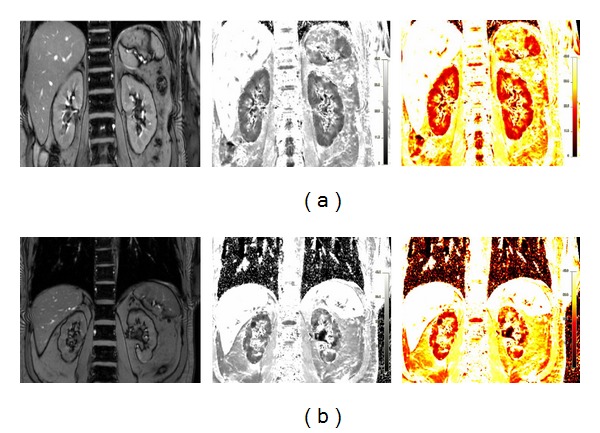
Example of blood oxygenation level-dependent MRI (BOLD-MRI) in a healthy volunteer (a) and in a patient suffering from chronic kidney disease (b). The anatomical templates are shown on the left side, the *R*2* maps in the middle, and the corresponding color maps on the right; low *R*2* levels (and supposed higher tissue oxygenation) in red and high *R*2* levels in yellow.

A relatively new technology called ‘Blood Oxygenation Level-Dependent Magnetic Resonance Imaging (BOLD-MRI), offers now possibilities to assess renal tissue oxygenation non-invasively in humans [25, 26]. The BOLD effect is based upon the different magnetic attributes of hemoglobin. Oxyhemoglobin is diamagnetic, whereas deoxyhemoglobin is paramagnetic. The magnetic field disturbances by paramagnetic molecules result in a loss of phase coherence, leading to signal attenuation on *T*2*-weighted MR images. Deoxygenated hemoglobin can therefore be used as an endogenous contrast agent. The ratio of deoxyhemoglobin to oxyhemoglobin concentrations is proportional to the pO_2_ of blood, and blood pO_2_ is supposed to be in equilibrium with the surrounding tissue. Hence, BOLD signal estimated by transverse relaxation rate *R*2* (=1/*T*2*) can be considered as a sensitive indicator of tissue pO_2_ [26]. The effect of the manipulation of the oxy- to deoxyhemoglobin ratio results in contrast changes in animals [27] and in humans [28]. *R*2* as measured by BOLD-MRI has been shown to correlate well with tissue pO_2_ [25, 29]. An example of MR images as obtained using the BOLD-MRI technique in a healthy volunteer is shown in Figure 1(a).

Since 1996, BOLD-MRI has been used in humans to evaluate, amongst others, the effect of water diuresis on renal medulla and cortex in healthy volunteers [34, 35] and diabetic patients [30] and to monitor changes in renal oxygenation after administration of different drugs [36, 37] and after acute renal ischemia [38, 39]. 

The BOLD-MRI technique itself has been criticized by some [40, 41], arguing that it is difficult to acquire the same anatomical slices in each participant when repeating the BOLD-MRI exams. However, the intraobserver variability of the cortical and medullary *R*2* values is low when performed by an experienced investigator and when using a standardized breath-hold technique [42]. An identical prehydration protocol and assessment of dietary sodium intake are at least as important, since both factors have been shown to influence the *R*2* signal (see below). 

## 4. Renal Tissue Oxygenation in Essential Hypertension

Essential hypertension is often characterized by an increased sodium retention, either in the proximal or distal nephron segments, a process requiring active sodium transport which might lead to increased tubular oxygen consumption [43, 44]. Besides, the renin-angiotensin system is in general activated in hypertensive patients, which may affect renal perfusion and oxygen consumption [45]. Therefore, essential hypertension could theoretically lead to cortical and medullary hypoxia. 

Indeed, studies in hypertensive rats using oxygen microelectrodes found pronounced medullary and cortical hypoxia in spontaneously hypertensive animals as compared to normotensive controls [14]. Most studies performed in humans so far have focused on renal artery stenosis, as discussed in more detail in another article of this number. Assessment of renal tissue oxygenation in patients with essential hypertension has only been performed in a few studies. In the first study, Textor et al. reported higher medullary *R*2* levels in 20 African-American hypertensive subjects, as compared to 20 Caucasians suggesting a lower medullary oxygenation in hypertensives [46]. This observation was explained by an increased renal medullary volume (measured by contrast-enhanced multidetector CT) per body surface area (BSA), coupled with increased sodium reabsorption and urinary prostaglandin F2 excretion in African Americans [47]. However, differences in age, body mass index (BMI), and estimated GFR (eGFR) hampered the ability to generalize these results. Besides, the study did not include a normotensive control group. 

Schachinger et al. showed that intravenous infusion of angiotensin II to six healthy volunteers leads to an acute rise in blood pressure (BP) and a simultaneous decrease of cortical *T*2*, suggesting a decrease in cortical oxygenation. Unfortunately, medullary oxygenation was not assessed in this study, and since hypertension was artificially induced, the results cannot be translated to the hypertensive population [48].

Finally, in our research unit, we have investigated ten young normotensive and eight untreated hypertensive men with BOLD-MRI after one week of a high sodium (>200 mmol/day) and again after one week of a low sodium diet (<100 mmol/day). Simultaneously, renal hemodynamics and renal sodium handling were assessed using, respectively, inulin-, PAH-, and endogenous lithium-clearances [47]. The main finding was that a low sodium intake was associated with an increased medullary oxygenation in both normo- and hypertensive individuals, independently of changes in renal perfusion. In our opinion, this is due to a salt depletion-induced shift of the oxygen-consuming sodium transport from the distal to the proximal tubules. Hence, the oxygen consumption in medulla and distal nephron was reduced, whereas oxygen consumption of the cortical proximal tubules increased under low sodium conditions. Cortical oxygenation was not altered, possibly due to the high cortical perfusion, and thus oxygen delivery, opposing the effect of changes in oxygen consumption. Indeed, increased proximal sodium reabsorption under low sodium conditions correlated positively with medullary oxygenation in normotensive individuals, yet not in hypertensive individuals. Hypertensive individuals had increased proximal sodium reabsorption under both low- and high-salt conditions, possibly explaining this lack of correlation and hence further confirming the role of the abnormal proximal reabsorption of sodium in hypertension [44]. 

Interestingly, at any sodium intake, hypertensive patients had slightly lower medullary *R*2* values than normotensive subjects, suggesting an increased rather than a decreased medullary oxygenation in young hypertensive men, again possibly due to reduced medullary oxygen consumption as a result of increased proximal sodium reabsorption. 

It is important to note that this study was performed in young hypertensive men, under extremely low or high sodium-intake conditions, and the oxygenation pattern might be different in older patients with hypertension-induced organ damage. 

Hence, acquired data in humans, so far, are not in line with animal studies and show no clear difference between normotensive and hypertensive subjects or even higher medullary oxygenation in hypertension as compared with normotensive controls. Whether this is due to less active medullar sodium transport, adaptations in medullar microcirculation or altered arterial venous shunting as compared to normal subjects merits further investigations. All presented data should be interpreted with caution, due to the small sample size, and confirmation in larger prospective studies is needed.

## 5. Renal Tissue Oxygenation in Chronic Kidney Disease

In clinical practice, renal ischemia has become a well-accepted cause of acute renal failure in situations of circulatory shock, accounting for almost 50% of cases of acute renal insufficiency [49]. However, the role of renal hypoxia in the pathophysiology of chronic kidney diseases remained largely underreported. Recently, Fine and Norman proposed that kidney injury leads to a vicious circle of tissue fibrosis, pursuant obliteration of the renal microvasculature, and further hypoxia [50]. Their statement was based on the fact that the extent of renal dysfunction is poorly associated with changes in glomerular morphology as seen in kidney biopsies of patients with chronic renal disease, whereas it correlates well with chronic tubulointerstitial fibrosis, a known consequence of renal hypoxia [51]. Once installed, the reduction of functional renal mass in CKD results in a number of common functional, structural, and metabolic adaptations that lead to further kidney damage, whatever the underlying cause [52]. Amongst these mechanisms are the activation of the renin-angiotensin system, glomerular hyperfiltration in residual glomeruli, the upregulation of inflammatory cytokines such as TGF-*β* which on his turn stimulates the formation of interstitial fibrosis, the development of proteinuria, and worsening of tissue hypoxia [52, 53].

Accumulating data from animal studies supports indeed, a pathogenic role of tissue hypoxia in the chronic deterioration of kidney function. The most convincing evidence probably comes from Manotham et al., who have shown in a remnant kidney model (which is a representative model of CKD) that the number of hypoxic tubules was markedly increased 4 and 7 days after subtotal (5/6) nephrectomy, as compared to a sham-operated control group [15]. Hypoxia was measured on kidney biopsies using immunostaining for pimonidazole. Of interest, these findings occurred in parallel with disturbed peritubular capillary perfusion and antedated any histologic evidence of tubulointerstitial damage. Hypoxia persisted until the development of interstitial fibrosis; in a subgroup treated with olmesartan, an angiotensin II type 1 receptor blocker, blockade of the renin-angiotensin system ameliorated peritubular capillary perfusion and tubular hypoxia and led to less interstitial fibrosis. Studies by Johnson et al. have also demonstrated that chronic systemic hypoxia causes renal interstitial damage and predisposes animals to persistent hypertension and its renal damages [54]. Medullary hypoxia has been documented in animal studies examining the development of diabetic nephropathy [55, 56]. 

BOLD-MRI can also be used to estimate renal tissue oxygenation in CKD patients (Figure 1(b)), and several studies have used BOLD-MRI in humans to investigate renal oxygenation in different forms of CKD. Diabetic nephropathy has been most frequently studied, and a summary of the results is shown in Table 1. Only one study reported lower medullary oxygenation in diabetics as compared with controls; two found no differences, whereas one even reported higher medullary oxygenation in diabetics. Concerning the cortex, again only the study of Yin et al. reported a lower cortical oxygenation (higher *R*2* levels) in diabetics as compared with controls [31]; the other studies found no differences. However, large differences existed in eGFR of the diabetics included in these studies. Moreover, different standardization procedures were followed (sober versus prehydration, medication withdrawal versus none), further hampering direct comparisons. In the study of Yin et al., diabetics were divided into four groups of increasing kidney damage (stage I–IV, according to Mogensen). Although medullary oxygenation was slightly lower in all diabetic groups as compared with controls, medullary oxygenation increased with worsening kidney function, despite the fact that hemoglobin levels strongly decreased, and age increased from stage I to IV. This unexpected finding was explained by the authors as a possible sign of decreased oxygen consumption due to reduced GFR and active tubular transport. This could suggest that in advanced CKD, reduced tubular oxygen consumption outlevels reduced medullary perfusion and oxygen diffusion and that progressive interstitial fibrosis merely increases rather than decreases medullary oxygenation. 

The largest study so far assessing renal oxygenation at different degrees of kidney dysfunction was recently reported by Michaely et al. [57]. This study included 400 patients who underwent MR imaging for nonspecific reasons, including staging of abdominal tumors and MR angiographies of intra-abdominal vessels. Of them, 280 patients had available serum creatinine values, and all KDOQI stages of CKD were represented. No correlation was found between *R*2* values and eGFR (according to the MDRD formula [58]), and *R*2* values were not affected by age and gender. Hence, despite some shortcomings of this study (no information on medication or dietary sodium intake, no standardization of BOLD and creatinine measurement), it seems that renal oxygenation is kept constant over a broad range of physiological and pathological conditions. As such, the findings of Michaely et al. put into question whether “chronic renal hypoxia” truly exists in humans or whether acute episodes of hypoxia are “corrected” by, for example, alterations in renal microcirculation and HIF-induced interstitial fibrosis. In line with this hypothesis, Juillard et al. have described acute increases in cortical and medullary *R*2* levels the first days after subtotal clipping of the renal artery [38]. Yet, four weeks after clipping, renal hypoxia could no longer be detected [59]. 

## 6. Antihypertensive Drugs and Renal Oxygenation

The use of blockers of the renin-angiotensin system (RAS blockers) as antihypertensive and antiproteinuric medication has been particularly effective in slowing the progression of renal disease in type 2 diabetics [60], and all existing guidelines advise to introduce an angiotensin converting enzymes inhibitor (ACEI) or an angiotensin II receptor blocker (ARB) in diabetic patients with CKD, as soon as microalbuminuria is detected or in case of hypertension [61].

Animal studies have suggested that administration of RAS blockers leads to an acute increase in renal tissue oxygenation [24, 53]. In humans, Djamali et al. reported a decrease in cortical *R*2* levels, suggesting increased oxygenation, in nine healthy volunteers two hours after the intake of 50 mg of losartan [62]. 

We recently performed a cross-over pilot study in twelve patients with type 2 diabetes and CKD (mean age 60 y, eGFR 62 mL ± 22 min/1.73 m²). Patients were either already on treatment with an ACEI or ARB or had a formal indication to start one (hypertension, (micro)albuminuria, or both). In this study, cortical and medullary *R*2* levels were not altered after one month of enalapril (20 mg qd) nor after one month of candesartan (16 mg qd) as compared with baseline (data presented at the 5th International meeting of the French Society of Hypertension, Paris, France, December 2011). Acute changes in *R*2* levels were not assessed in this study. However, in combination with the study of Djamali et al., one might conclude that RAS blockers induce acute yet not chronic increases in renal oxygenation.

The administration of furosemide has been shown to induce in an acute drop of medullary and to a lesser degree cortical *R*2* levels in healthy volunteers, in diabetics, and in patients with renal artery stenosis suggesting a diuretic-induced increase in renal oxygenation [21, 46]. The effect of furosemide on renal oxygenation has been attributed to a reduction of the active oxygen-consuming sodium transport in the ascending loop of Henle [46]. To the best of our knowledge, no studies have assessed the effect of chronic furosemide intake on renal oxygenation.

## 7. Conclusions and Perspectives 

Taken together, the BOLD-MRI technique has opened an exciting new field of research that allows for the first time the assessment of renal oxygenation non-invasively in humans. The changes in renal oxygenation observed in response to furosemide or in association with dietary sodium intake suggest that renal sodium handling is one of the main determinants of renal tissue oxygenation. 

 So far, no studies have convincingly demonstrated that renal oxygenation is reduced in essential hypertension, diabetes, or CKD. These findings are in contrast with most animal studies. First of all, it might be that BOLD-MRI is not sensitive enough or simply not as good a tool to assess renal oxygenation. Indeed, the assumption that tissue oxygenation varies with blood oxygenation might not always hold in kidneys that are characterized by profound arteriovenous oxygen shunting. Nonetheless, several animal studies have validated this technique. Another possibility is that animal studies provide excellent models to study short-term changes in oxygenation but that they are suboptimal in simulating the long-term changes and adaptations in the kidney that might have occurred after several decades of exposure to chronic disease states such as diabetes or hypertension. Whatever the truth may be, BOLD-MRI and other new radiological techniques that study renal functioning non-invasively merit the full attention of clinicians, and their development will certainly help to further unravel the role of renal oxygenation in the development and progression of essential hypertension and CKD in humans.

## Figures and Tables

**Table 1:** Case control studies that have assessed renal oxygenation in diabetics.

Study	Patient	*N*	Age (y)	eGFR (mL/min/1.73 m²)	MRI (Tesla)	Medullary *R*2* (1/sec)	Cortical *R*2* (1/sec)
Epstein et al. (2002) [30]	DM2	9	48 ± 3	119 ± 6	1.5	17 ± 0.5	13 ± 0.2
	Control	9	51 ± 2	133 ± 7	1.5	17 ± 0.8	13 ± 0.2
Yin et al. (2012) [31]	DM2	48	38–70	8–110	3	Higher	Higher
	Control	67	51 ± 14	n.m.	3	Lower	Lower
Wang et al. (2011) [32]	DM2	20	65 (49–77)	47 (14–71)	1.5	14 ± 2	11 ± 1
	Control	7	35 (30–45)	n.m.	1.5	19 ± 1	12 ± 1
Inoue et al. (2011) [33]	DM2	43	59 ± 11	44 ± 28	1.5	n.m.	74 ± 8^a^
	Control	10	36	n.m.	1.5	59 ± 5	74 ± 5^a^

^a^ *T*2* value instead of *R*2* value reported; n.m.: not mentioned.

## References

[1] van den Hoogen PCW, Feskens EJM, Nagelkerke NJD (2000). The relation between blood pressure and mortality due to coronary heart disease among men in different parts of the world. *The New England Journal of Medicine*.

[2] Flack JM, Neaton J, Grimm R (1995). Blood pressure and mortality among men with prior myocardial infarction. *Circulation*.

[3] Klag MJ, Whelton PK, Randall BL (1996). Blood pressure and end-stage renal disease in men. *The New England Journal of Medicine*.

[4] Brancati FL, Whelton PK, Kuller LH, Klag MJ (1996). Diabetes mellitus, race, and socioeconomic status. A population-based study. *Annals of Epidemiology*.

[5] Stengel B, Billon S, van Dijk PCW (2003). Trends in the incidence of renal replacement therapy for end-stage renal disease in Europe, 1990–1999. *Nephrology Dialysis Transplantation*.

[6] Glassock RJ (2004). The rising tide of end-stage renal disease: what can be done?. *Clinical and Experimental Nephrology*.

[7] Weisstuch JM, Dworkin LD (1992). Does essential hypertension cause end-stage renal disease. *Kidney International*.

[8] Jamerson KA, Townsend RR (2011). The attributable burden of hypertension: focus on CKD. *Advances in Chronic Kidney Disease*.

[9] Schrier RW, Masoumi A, Elhassan E (2010). Aldosterone: role in edematous disorders, hypertension, chronic renal failure, and metabolic syndrome. *Clinical Journal of the American Society of Nephrology*.

[10] Coffman TM, Himmelstein S, Best C, Klotman PE (1989). Post-transplant hypertension in the rat: effects of captopril and native nephrectomy. *Kidney International*.

[11] Rettig R (1993). Does the kidney play a role in the aetiology of primary hypertension? Evidence from renal transplantation studies in rats and humans. *Journal of Human Hypertension*.

[12] Curtis JJ, Luke RG, Diethelm AG (1985). Benefits of removal of native kidneys in hypertension after renal transplantation. *The Lancet*.

[13] Nangaku M (2006). Chronic hypoxia and tubulointerstitial injury: a final common pathway to end-stage renal failure. *Journal of the American Society of Nephrology*.

[14] Welch WJ, Baumgärtl H, Lübbers D, Wilcox CS (2001). Nephron pO_2_ and renal oxygen usage in the hypertensive rat kidney. *Kidney International*.

[15] Manotham K, Tanaka T, Matsumoto M (2004). Evidence of tubular hypoxia in the early phase in the remnant kidney model. *Journal of the American Society of Nephrology*.

[16] Aukland K, Krog J (1960). Renal oxygen tension. *Nature*.

[17] Schurek HJ, Jost U, Baumgartl H, Bertram H, Heckmann U (1990). Evidence for a preglomerular oxygen diffusion shunt in rat renal cortex. *American Journal of Physiology*.

[18] Kiil F (1977). Renal energy metabolism and regulation of sodium reabsorption. *Kidney International*.

[19] Brezis M, Rosen S, Silva P, Epstein FH (1984). Renal ischemia: a new perspective. *Kidney International*.

[20] Welch WJ (2006). Intrarenal oxygen and hypertension. *Clinical and Experimental Pharmacology and Physiology*.

[21] Gomez SI, Warner L, Haas JA (2009). Increased hypoxia and reduced renal tubular response to furosemide detected by BOLD magnetic resonance imaging in swine renovascular hypertension. *American Journal of Physiology*.

[22] Prchal JT (2003). Delivery on demand—a new era of gene therapy?. *The New England Journal of Medicine*.

[23] Nangaku M, Eckardt KU (2007). Hypoxia and the HIF system in kidney disease. *Journal of Molecular Medicine*.

[24] Norman JT, Stidwill R, Singer M, Fine LG (2003). Angiotensin II blockade augments renal cortical microvascular pO_2_ indicating a novel, potentially renoprotective action. *Nephron*.

[25] Prasad PV, Edelman RR, Epstein FH (1996). Noninvasive evaluation of intrarenal oxygenation with BOLD MRI. *Circulation*.

[26] Prasad PV (2006). Evaluation of intra-renal oxygenation by BOLD MRI. *Nephron*.

[27] Ogawa S, Lee TM, Nayak AS, Glynn P (1990). Oxygenation-sensitive contrast in magnetic resonance image of rodent brain at high magnetic fields. *Magnetic Resonance in Medicine*.

[28] Kwong KK, Belliveau JW, Chesler DA (1992). Dynamic magnetic resonance imaging of human brain activity during primary sensory stimulation. *Proceedings of the National Academy of Sciences of the United States of America*.

[29] Pedersen M, Dissing TH, Morkenborg J (2005). Validation of quantitative BOLD MRI measurements in kidney: application to unilateral ureteral obstruction. *Kidney International*.

[30] Epstein FH, Veves A, Prasad PV (2002). Effect of diabetes on renal medullary oxygenation during water diuresis. *Diabetes Care*.

[31] Yin WJ, Liu F, Li XM (2012). Noninvasive evaluation of renal oxygenation in diabetic nephropathy by BOLD-MRI. *European Journal of Radiology*.

[32] Wang ZJ, Kumar R, Banerjee S, Hsu CY (2011). Blood oxygen level-dependent (BOLD) MRI of diabetic nephropathy: preliminary experience. *Journal of Magnetic Resonance Imaging*.

[33] Inoue T, Kozawa E, Okada H (2011). Noninvasive evaluation of kidney hypoxia and fibrosis using magnetic resonance imaging. *Journal of the American Society of Nephrology*.

[34] Prasad PV, Epstein FH (1999). Changes in renal medullary pO_2_ during water diuresis as evaluated by blood oxygenation level-dependent magnetic resonance imaging: effects of aging and cyclooxygenase inhibition. *Kidney International*.

[35] Epstein FH, Prasad P (2000). Effects of furosemide on medullary oxygenation in younger and older subjects. *Kidney International*.

[36] Hofmann L, Simon-Zoula S, Nowak A (2006). BOLD-MRI for the assessment of renal oxygenation in humans: acute effect of nephrotoxic xenobiotics. *Kidney International*.

[37] Kristensen DH, Pedersen M, Grøn MC (2005). Intrarenal blood oxygenation and renal function measured by magnetic resonance imaging during long-term cyclosporine treatment. *Transplantation Proceedings*.

[38] Juillard L, Lerman LO, Kruger DG (2004). Blood oxygen level-dependent measurement of acute intra-renal ischemia. *Kidney International*.

[39] Alford SK, Sadowski EA, Unal O (2005). Detection of acute renal ischemia in swine using blood oxygen level-dependent magnetic resonance imaging. *Journal of Magnetic Resonance Imaging*.

[40] Thelwall PE, Taylor R, Marshall SM (2011). Non-invasive investigation of kidney disease in type 1 diabetes by magnetic resonance imaging. *Diabetologia*.

[41] Zuo CS, Rofsky NM, Mahallati H (2003). Visualization and quantification of renal R2* changes during water diuresis. *Journal of Magnetic Resonance Imaging*.

[42] Simon-Zoula SC, Hofmann L, Giger A (2006). Non-invasive monitoring of renal oxygenation using BOLD-MRI: a reproducibility study. *NMR in Biomedicine*.

[43] Chiolero A, Maillard M, Nussberger J, Brunner HR, Burnier M (2000). Proximal sodium reabsorption: an independent determinant of blood pressure response to salt. *Hypertension*.

[44] Burnier M, Bochud M, Maillard M (2006). Proximal tubular function and salt sensitivity. *Current Hypertension Reports*.

[45] Deng A, Miracle CM, Suarez JM (2005). Oxygen consumption in the kidney: effects of nitric oxide synthase isoforms and angiotensin II. *Kidney International*.

[46] Textor SC, Glockner JF, Lerman LO (2008). The use of magnetic resonance to evaluate tissue oxygenation in renal artery stenosis. *Journal of the American Society of Nephrology*.

[47] Pruijm M, Hofmann L, Maillard M (2010). Effect of sodium loading/depletion on renal oxygenation in young normotensive and hypertensive men. *Hypertension*.

[48] Schachinger H, Klarhöfer M, Linder L, Drewe J, Scheffler K (2006). Angiotensin II decreases the renal MRI blood oxygenation level-dependent signal. *Hypertension*.

[49] Thadhani R, Pascual M, Bonventre JV (1996). Acute renal failure. *The New England Journal of Medicine*.

[50] Fine LG, Norman JT (2008). Chronic hypoxia as a mechanism of progression of chronic kidney diseases: from hypothesis to novel therapeutics. *Kidney International*.

[51] Eddy AA (2005). Progression in chronic kidney disease. *Advances in Chronic Kidney Disease*.

[52] Abboud H, Henrich WL (2010). Clinical practice. Stage IV chronic kidney disease. *The New England Journal of Medicine*.

[53] Deng A, Tang T, Singh P (2009). Regulation of oxygen utilization by angiotensin II in chronic kidney disease. *Kidney International*.

[54] Johnson RJ, Rodriguez-Iturbe B, Nakagawa T, Kang DH, Feig DI, Herrera-Acosta J (2005). Subtle renal injury is likely a common mechanism for salt-sensitive essential hypertension. *Hypertension*.

[55] dos Santos EA, Li LP, Ji L, Prasad PV (2007). Early changes with diabetes in renal medullary hemodynamics as evaluated by fiberoptic probes and BOLD magnetic resonance imaging. *Investigative Radiology*.

[56] Palm F, Friederich M, Carlsson PO, Hansell P, Teerlink T, Liss P (2008). Reduced nitric oxide in diabetic kidneys due to increased hepatic arginine metabolism: implications for renomedullary oxygen availability. *American Journal of Physiology*.

[57] Michaely HJ, Metzger L, Haneder S, Hansmann J, Schoenberg SO, Attenberger UI (2012). Renal BOLD-MRI does not reflect renal function in chronic kidney disease. *Kidney International*.

[58] Levey AS, Coresh J, Greene T (2006). Using standardized serum creatinine values in the modification of diet in renal disease study equation for estimating glomerular filtration rate. *Annals of Internal Medicine*.

[59] Rognant N, Guebre-Egziabher F, Bacchetta J (2011). Evolution of renal oxygen content measured by BOLD MRI downstream a chronic renal artery stenosis. *Nephrology Dialysis Transplantation*.

[60] Pohl MA, Blumenthal S, Cordonnier DJ (2005). Independent and additive impact of blood pressure control and angiotensin II receptor blockade on renal outcomes in the irbesartan diabetic nephropathy trial: clinical implications and limitations. *Journal of the American Society of Nephrology*.

[61] Khan NA, Hemmelgarn B, Herman RJ (2009). The 2009 Canadian Hypertension Education Program recommendations for the management of hypertension: part 2—therapy. *The Canadian Journal of Cardiology*.

[62] Djamali A, Sadowski EA, Muehrer RJ (2007). BOLD-MRI assessment of intrarenal oxygenation and oxidative stress in patients with chronic kidney allograft dysfunction. *American Journal of Physiology*.

